# The predictive value of ICD-10 diagnostic coding used to assess Charlson comorbidity index conditions in the population-based Danish National Registry of Patients

**DOI:** 10.1186/1471-2288-11-83

**Published:** 2011-05-28

**Authors:** Sandra K Thygesen, Christian F Christiansen, Steffen Christensen, Timothy L Lash, Henrik T Sørensen

**Affiliations:** 1 Department of Clinical Epidemiology, The Institute of Clinical Medicine, Aarhus University Hospital, Denmark; 2 Department of Epidemiology, Boston University School of Public Health, USA

## Abstract

**Background:**

The Charlson comorbidity index is often used to control for confounding in research based on medical databases. There are few studies of the accuracy of the codes obtained from these databases.

We examined the positive predictive value (PPV) of the ICD-10 diagnostic coding in the Danish National Registry of Patients (NRP) for the 19 Charlson conditions.

**Methods:**

Among all hospitalizations in Northern Denmark between 1 January 1998 and 31 December 2007 with a first-listed diagnosis of a Charlson condition in the NRP, we selected 50 hospital contacts for each condition. We reviewed discharge summaries and medical records to verify the NRP diagnoses, and computed the PPV as the proportion of confirmed diagnoses.

**Results:**

A total of 950 records were reviewed. The overall PPV for the 19 Charlson conditions was 98.0% (95% CI; 96.9, 98.8). The PPVs ranged from 82.0% (95% CI; 68.6%, 91.4%) for diabetes with diabetic complications to 100% (one-sided 97.5% CI; 92.9%, 100%) for congestive heart failure, peripheral vascular disease, chronic pulmonary disease, mild and severe liver disease, hemiplegia, renal disease, leukaemia, lymphoma, metastatic tumour, and AIDS.

**Conclusion:**

The PPV of NRP coding of the Charlson conditions was consistently high.

## Background

Comorbidities are coexistent diseases to a disease of interest [1] or index disease [2]. Comorbidities may directly affect the prognosis of the disease of interest, or may indirectly affect the prognosis by affecting the choice of treatment [1-3].

An index of comorbidity level has the advantage of bringing several comorbidities into a single numeric score, thereby reducing the number of candidate variables into a manageable set of proxy variables, which is especially beneficial when using administrative databases or medical registries [4,5]. The Charlson comorbidity index is the most widely used comorbidity index [3,6]. It was developed to predict 1-year mortality among 604 medical patients based on comorbidity data obtained from hospital chart review in a single US hospital in 1984 [6]. The 19 Charlson conditions were selected and weighted according to their potential influence on mortality and validated for predicting 1-year mortality in a cohort of 685 breast cancer patients [6]. Since then, the Charlson comorbidity index has been adapted for use with data from administrative databases and medical registries that record medical conditions using the International Classification of Diseases, 9^th ^revision (ICD-9), a Clinical Modification of ICD-9 (ICD-9-CM) [7,8], and recently also the International Classification of Diseases, 10^th ^revision (ICD-10) [9-11].

Only two studies have examined the accuracy of diagnosis coding of Charlson comorbidity conditions in administrative hospital registries compared with diagnoses obtained through medical records. A study by Quan et al [12] was carried out in 1996-1997 in Calgary, Canada using ICD-9 diagnosis codes, but the results may not necessarily hold today for European registries using ICD-10 codes. Henderson et al. assessed the quality of coding in Victoria, Australia soon after the implementation of ICD-10 in 1998-2001 compared with ICD-9 coding in the earlier years and found high coding quality in both time periods [13].

Medical registries and administrative databases offer an important resource for studies of public health issues [14]. Scandinavian population-based medical registries can be linked using unique personal identifiers and are therefore used extensively for epidemiologic research [15,16]. Data collection and coding procedures may vary across countries [17], and no Nordic study has validated the coding procedure on all conditions included in the Charlson comorbidity index. Measuring comorbidity accurately is important, since controlling for confounding by comorbidity affects the validity of such epidemiologic studies [3,18]. Sufficient control for confounding requires high data quality [19].

We therefore conducted this study to assess the positive predictive value (PPV) of the coding of each of the 19 Charlson comorbidity conditions assessed by ICD-10 diagnoses from a population-based medical registry.

## Methods

### Study population

This study was conducted in the North Jutland Region, Denmark using patients with diagnoses registered in the Danish National Registry of Patients (NRP) between 1 January 1998 and 31 December 2007. The population of this region is approximately 500,000 people, corresponding to about 11% of the total Danish population. The Danish population receives tax-supported health care without additional charge.

### The National Registry of Patients

**Figure 1:**
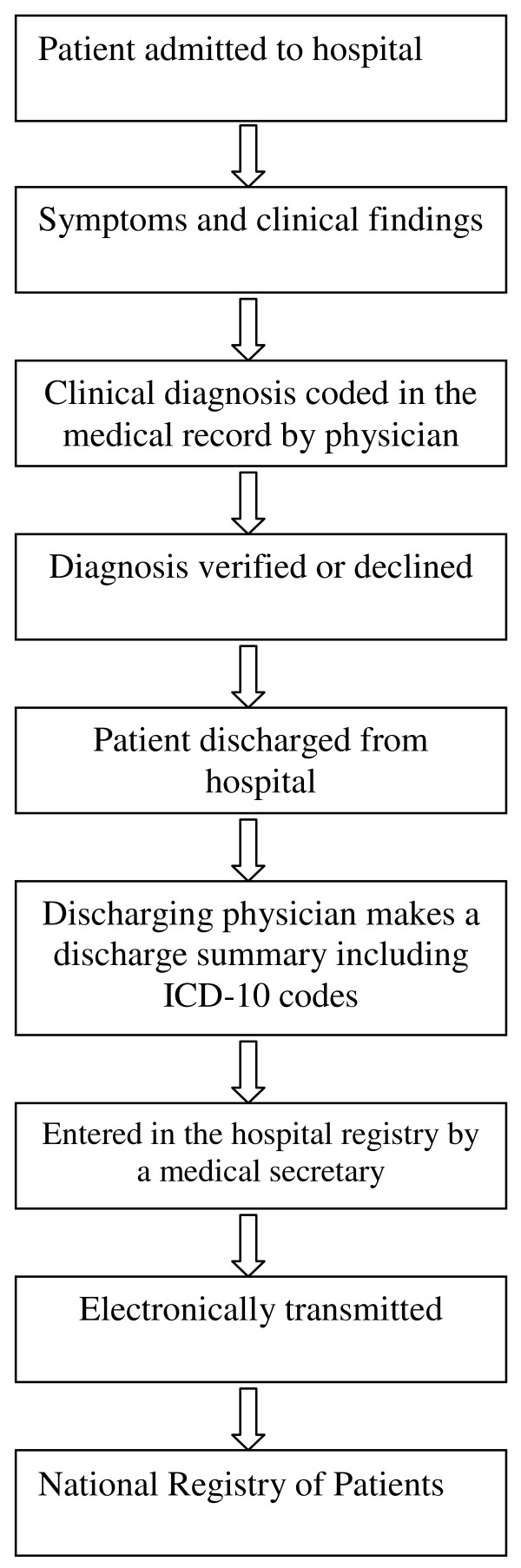
**Coding process during an admission to a hospital in Denmark**.

The Danish NRP includes data on all non-psychiatric hospital admissions in Denmark since 1977 and outpatient clinic and emergency room visits since 1995. The NRP includes data on date of admissions and discharges, surgical procedures performed, and up to 20 diagnoses classified according to the International Classification of Diseases, 8^th ^revision (ICD-8) until the end of 1993 and 10^th ^revision (ICD-10) thereafter. The physician who discharges the patient reviews the medical record and makes a discharge summary including discharge diagnoses coded using ICD codes. ICD codes are then entered by the medical secretary into the hospital registry. From there, the data are electronically transmitted to the NRP at the National Board of Health (Figure 1) [16,20].

We used the NRP to identify all hospital contacts (comprising both in- and outpatients) in the study population with one of the Charlson comorbidity conditions as a first-listed diagnosis [21]. We did not make any age restriction. Outpatients included emergency room contacts and patients followed in outpatient clinics. According to Danish practice, the first-listed diagnosis in the discharge record is the main reason for the hospital contact. For each of the 19 Charlson comorbidity conditions, we selected randomly five hospital contacts per year in the ten year study period, yielding a total of 950 hospital contacts.

We used the patient's unique personal identification number and date of admission and discharge to identify the discharge summary for each of the 950 hospital contacts. The discharge summary describes the most important events during the hospital contact, including reason for admission, diagnostic work-up, treatment, prescribed medications, and plan after discharge [22]. All discharge summaries were reviewed by the same physician (SKT).

### Record review

The review of the discharge summary and medical record began with a confirmation of the personal identification number and the date of admission and discharge for the hospital contact retrieved from the NRP. We then proceeded with confirmation of the diagnosis. We considered a diagnosis to be confirmed if the discharge summary described the exact diagnosis or a diagnosis within the same Charlson comorbidity condition. For example, if a discharge summary or medical record indicated non-insulin dependent diabetes mellitus, and this disease was coded as insulin dependent diabetes mellitus in the NRP, then the diagnosis was considered to be confirmed because the Charlson comorbidity index does not distinguish between non-insulin dependent and insulin dependent diabetes mellitus. If the diagnosis was not described in the discharge summary or if the discharge summary was not available, then the medical record was reviewed to determine whether the diagnosis code could be confirmed. Discharge summaries from outpatient clinic contacts may only include a description of treatment, and in these cases the medical record was retrieved. When the reviewing physician had any doubt about whether the discharge summary or medical record agreed with the NRP ICD-10 code, the discharge summary or medical record was reviewed by a second physician (CFC), and the two physicians reached a consensus agreement. The review process was conducted twice for all patients by SKT (second review was done to check for typing errors).

### Statistical analysis

We assessed the accuracy of the ICD-10 diagnostic codes in the NRP by comparison with the discharge summary or medical record, which were considered the reference standard. We quantified the accuracy by computing the positive predictive value and its corresponding 95% confidence intervals (CI), calculated with Clopper-Pearson binomial confidence intervals [23]. The positive predictive value was the proportion of Charlson comorbidity conditions identified in the record contacts collected from the NRP that could be confirmed in the discharge summary or in the medical record.

We stratified the analyses by age, sex, and inpatients and outpatients both separately and together to elucidate any differences in the PPV. We also report the proportion of cases for whom the medical record was retrieved, for both inpatients and outpatients.

Data were entered in EpiData (EpiData Association, Odense, Denmark, http://www.epidata.dk) and analysed with STATA version 9.2 (StataCorp, College Station, Texas, USA). The study was approved by The Danish Data Protection Agency (record number: 2006-53-1396)

## Results

From the 950 diagnoses codes from the NRP we identified all 950 hospital contacts. Of these 588 (61.9%) were inpatients and 362 (38.1%) were outpatients. 65 (6.8%) patients were younger than 18 years of age. Of those most had leukemia (20 patients) or hemiplegia (16 patients) and the rest included diabetes mellitus, chronic pulmonary disease, connective tissue disease, moderate to severe renal disease, AIDS, lymphoma and any tumor. Discharge summaries needed more information to confirm the registry diagnosis in 238 (25%) of the contacts so the medical record was reviewed for these.

The overall positive predictive value for the first-listed diagnosis included in the 19 Charlson comorbidity conditions was 98.0% (95% CI; 96.9%, 98.8%). The PPV for the first-listed diagnoses in each of the Charlson comorbidity conditions ranged from 82.0% (95% CI; 68.6%, 91.4%) for diabetes mellitus with diabetic complications to 100% (one-sided 97.5% CI 92.2%, 100%) for congestive heart failure, peripheral vascular disease, chronic pulmonary disease, mild and severe liver disease, hemiplegia, renal disease, leukaemia, lymphoma, metastatic tumour and AIDS (Table 1). We found virtually no differences when stratifying in- and outpatients by each of the Charlson comorbidity condition.

**Table 1:** Positive predictive value (PPV) of the National Registry of Patients (NRP) first-listed ICD codes for the Charlson comorbidities

Charlson condition	Corresponding International Classification of Diseases, 10^th ^revision codes	Number of correct coded contacts/number reviewed	PPV % (95% CI)* First-listed diagnoses
All Charlson conditions		931/950	98.0 (96.9 - 98.8)

Myocardial infarction	I21;I22;I23	49/50	98.0 (89.4 - 99.9)

Congestive heart failure	I50; I11.0; I13.0; I13.2	50/50	100 (92.9 - 100)

Peripheral vascular disease	I70; I71; I72; I73; I74; I77	50/50	100 (92.9 - 100)

Cerebrovascular disease	I60-I69; G45; G46	47/50	94.0 (83.5 - 98.7)

Dementia	F00-F03; F05.1; G30	49/50	98.0 (89.4 - 99.9)

Chronic pulmonary disease	J40-J47; J60-J67; J68.4; J70.1;	50/50	100 (92.9 - 100)
	J70.3; J84.1; J92.0; J96.1; J98.2; J98.3		

Connective tissue disease	M05; M06; M08; M09;M30;M31;	49/50	98.0 (89.4 - 99.9)
	M32; M33; M34; M35; M36; D86		

Ulcer disease	K22.1; K25-K28	49/50	98.0 (89.4 - 99.9)

Mild liver disease	B18; K70.0-K70.3; K70.9; K71; K73; K74; K76.0	50/50	100 (92.9 - 100)

Diabetes mellitus	E10.0, E10.1; E10.9	48/50	96.0 (86.3 - 99.5)
	E11.0; E11.1; E11.9		

Hemiplegia	G81; G82	50/50	100 (92.9 - 100)

Moderate/severe renal disease	I12; I13; N00-N05; N07; N11; N14; N17-N19; Q61	50/50	100 (92.9 - 100)

Diabetes mellitus with chronic complications	E10.2-E10.8	41/50	82.0 (68.6 - 91.4)
	E11.2-E11.8		

Any tumor	C00-C75	49/50	98.0 (89.4 - 99.9)

Leukemia	C91-C95	50/50	100 (92.9 - 100)

Lymphoma	C81-C85; C88; C90; C96	50/50	100 (92.9 - 100)

Moderate/severe liver disease	B15.0; B16.0; B16.2; B19.0; K70.4; K72; K76.6; I85	50/50	100 (92.9 - 100)

Metastatic solid tumor	C76-C80	50/50	100 (92.9 - 100)

AIDS	B21-B24	50/50	100 (92.9 - 100)

*When the PPV is 100%, a one-sided 97.5% confidence interval was estimated.

When estimating the coding accuracy according to each stratum, we found a PPV of 98.6 (95% CI; 97.3%, 99.4%) for inpatients and 97.0 (95% CI; 94.6%, 98.5%) for outpatients. In females the PPV was 98.2% (95% CI; 96.5%, 99.2%) and in males 97.8% (95% CI; 96.1%, 98.9%). The PPVs were 100% (one-sided 97.5% CI; 94.5%, 100%) for patients aged below 18 years, 97.4% (95% CI; 94.0%, 99.1%) for patients aged 18 to 49 years, 97.3% (95% CI; 94.4%, 98.9%) for patients aged 50 to 64 years, 99.0% (95% CI; 96.5%, 99.9%) for patients aged 65 to 74 years, and 97.9% (95% CI; 95.1%, 99.3%) for patients 75 years or older.

The medical record review was required for confirmation of the diagnosis in 36.2% of outpatients, but only 18.2% of inpatients.

## Discussion

We found a positive predictive value greater than 90% for almost all ICD-10 diagnostic codes used to ascertain the Charlson comorbidity conditions in the NRP. This accuracy is better than the accuracy reported in earlier studies [12,13].

Our study was conducted in an area with virtually complete data from the population-based administrative registries on all hospitalized patients during the study period. We examined patients admitted to hospitals in one region in Denmark, but do not expect that rates of coding errors would differ across the regions. We sampled the same number of discharge summaries and medical records from each of the ten years of the study period, and the NRP data were of excellent quality throughout the time period. We validated the 19 conditions included in the Charlson comorbidity index, which were selected by Charlson because they were important predictors of one-year mortality risk. This set of conditions, therefore, contains serious diseases that are readily diagnosed. The excellent PPV of these diagnostic codes in the NRP may not apply to less severe conditions recorded in the NRP. Furthermore, wrong coded diagnoses within the same Charlson category were considered confirmed, as errors of this type would not affect the Charlson comorbidity index score. Finally, we verified the discharge physicians' coding compared with the description in the discharge summary or medical record (Figure 1), but did not examine whether the diagnostic criteria were actually fulfilled.

We found almost twice as many outpatients as inpatients, who needed the entire medical journal to be retrieved for confirmation of the registry recorded diagnosis. The reason was that the physician often continued writing in the record from a previous visit. Therefore it was more often a short note, answers on blood tests etc. and often the diagnosis was not mentioned. Sometimes a note just showed that the patient missed his/her appointment.

The diagnosis code was known to the physician reviewer before reviewing the discharge summary. When in doubt, this may affect the judgement of the diagnostic coding in favour of a confirmation and we cannot rule this out as a possible partial explanation of the high PPVs. We have no information on patients with a Charlson comorbidity condition that was not diagnosed at a hospital; however, this concern is unlikely to influence our results because patients with these serious diseases are likely to have had previous hospital contact. Because we do not have un-coded patients, fx. a patient with diabetes hospitalized with a pneumonia may not always be coded with diabetes, we are unable to estimate the negative predictive value, sensitivity, or specificity, which are also important measures of validity of administrative hospital discharge databases [24].

Diabetes mellitus with diabetic complications had the lowest PPV, mainly because the diabetic complications could not be confirmed in the discharge summary or the medical record. For example, dysregulation of diabetes was typically interpreted as a complication without specification. A previous Danish study found a higher PPV of diabetes registration (insulin dependent diabetes mellitus of 96.3 (95% CI; 95.5-97.2) and noninsulin dependent diabetes mellitus of 97.9% (95% CI; 97.2; 98.5)) [25], however, they did not study the misclassification of the complications related to diabetes mellitus.

Two earlier studies have validated the quality of diagnostic coding in administrative databases used to ascertain Charlson comorbidities [12,13] and one validated the coding process according to the ICD-10-AM. Henderson et al [13] assessed the quality of coding in routinely collected hospital discharge data in Australia based on ICD-10-AM for 1998-1999 (n = 7,004) and in 2000-2001 (n = 7,631). Their PPV (2000-2001) ranged from 62% (95% CI; 48, 76) for peripheral vascular disease to 94% (95% CI; 91, 97) for metastatic cancer. Their PPV for HIV was not included, as there were no prevalent cases. The validation study was completed shortly after the implementation of ICD-10 to examine whether the data quality was maintained or improved. Our study started 4 years after the implementation of ICD-10, which may explain our consistently higher PPVs.

The Canadian study [12] validated the quality of diagnostic coding on administrative data using the ICD-9-CM diagnoses included in the Charlson comorbidity index. The study was conducted in 1996-1997 including 1,200 inpatients in Calgary, Alberta. They found a PPV ranging from 44.0% to 96.3%. Some of the Charlson comorbidities were associated with considerable coding errors and were based on ICD-9-CM, which is not as widely used as the ICD-10. PPV values were found lower than 50% for liver disease (both mild and moderate to severe) and rheumatologic diseases.

Humphries et al [26] validated 7 of the Charlson comorbidities in 817 patients undergoing percutaneous coronary intervention at a single hospital from 1994-1995. The study was based on ICD-9-CM and their PPV ranged from 50.6% to 93.3% using the patient chart review data as the reference standard.

Several Danish studies have estimated the PPV of selected diagnoses included in the Charlson comorbidity index (e.g. acute myocardial infarction [27], cerebrovascular disease [28,29], dementia [30], rheumatoid arthritis [31], liver cirrhosis [32], diabetes mellitus [25], cancer [33], haematological malignancies [34] and HIV [35]) in the NRP and generally report of lower PPV's than in our study. These studies validated diagnoses using strict diagnostic criteria, requiring for example specific clinical investigations. If these specific diagnostic criteria were not satisfied, then a patient was classified as not having the disease, even if the physician had diagnosed and treated the patient for that disease. These other studies were therefore meant to validate diagnoses, whereas we were validating ICD-10 codes against the diagnosis assigned by the treating physician. The difference in objective may explain the lower accuracies reported in these other Danish studies.

## Conclusion

The PPV of diagnosis coding in the Danish NRP for conditions included in the Charlson comorbidity index is very high. The high positive predictive value in our study suggests that the NRP ICD-10 diagnostic codes are coded very accurately in comparison to the discharge physician's diagnosis. We could not, however, confirm whether the diagnosis was correct. The high accuracy supports the use of ICD-10 codes in future research to control for confounding by comorbidity as measured by the Charlson comorbidity index.

## List of abbreviations

ICD-8: International Classification of Diseases, 8^th ^revision; ICD-9: International Classification of Diseases, 9^th ^revision; ICD-10: International Classification of Diseases, 10^th ^revision; NRP: National Registry of Patients; PPV: Positive predictive value; CI: Confidence intervals; SKT: Sandra Kruchov Thygesen; CFC: Christian Fynbo Christiansen

## Competing interests

Declaration of funding interests: This study was funded by the Clinical Epidemiological Research Foundation ('Klinisk Epidemiologisk Forskningsfond'), Denmark. The Department of Clinical Epidemiology, Aarhus University Hospital, receives funding for other studies from companies in the form of research grants (and administered by) Aarhus University. None of these studies have any relation to the present study.

Declaration of personnel interests: Author and co-authors have no conflict of interest.

## Authors' contributions

Study concept and design: SKT, CFC and HTS. Analysis and interpretation of data: SKT, CFC, SC, TLL and HTS. Drafting of the manuscript: SKT, CFC and SC. Statistical analysis: SKT. Critical revision of the manuscript for important intellectual content: CFC, SC, TLL and HTS. Study supervision: CFC, SC and HTS. All authors approved the final version.

## Pre-publication history

The pre-publication history for this paper can be accessed here:

http://www.biomedcentral.com/1471-2288/11/83/prepub
